# Research on Dual-Phase Composite Forming Process and Platform Construction of Radial Gradient Long Bone Scaffold

**DOI:** 10.3390/bioengineering11090869

**Published:** 2024-08-27

**Authors:** Haiguang Zhang, Rui Wang, Yongteng Song, Yahao Wang, Qingxi Hu

**Affiliations:** 1 Rapid Manufacturing Engineering Center, School of Mechatronic Engineering and Automation, Shanghai University, Shanghai 200444, China; 2 Shanghai Key Laboratory of Intelligent Manufacturing and Robotics, Shanghai University, Shanghai 200072, China; 3 National Demonstration Center for Experimental Engineering Training Education, Shanghai University, Shanghai 200444, China

**Keywords:** radial gradient tapering, bone scaffold, continuous printing

## Abstract

The structure and composition of natural bone show gradient changes. Most bone scaffolds prepared by bone tissue engineering with single materials and structures present difficulties in meeting the needs of bone defect repair. Based on the structure and composition of natural long bones, this study proposed a new bone scaffold preparation technology, the dual-phase composite forming process. Based on the composite use of multiple biomaterials, a bionic natural long bone structure bone scaffold model with bone scaffold pore structure gradient and material concentration gradient changes along the radial direction was designed. Different from the traditional method of using multiple nozzles to achieve material concentration gradient in the scaffold, the dual-phase composite forming process in this study achieved continuous 3D printing preparation of bone scaffolds with gradual material concentration gradient by controlling the speed of extruding materials from two feed barrels into a closed mixing chamber with one nozzle. Through morphological characterization and mechanical property analysis, the results showed that BS-G (radial gradient long bone scaffolds prepared by the dual-phase composite forming process) had obvious pore structure gradient changes and material concentration gradient changes, while BS-T (radial gradient long bone scaffolds prepared by printing three concentrations of material in separate regions) had a discontinuous gradient with obvious boundaries between the parts. The compressive strength of BS-G was 1.00 ± 0.19 MPa, which was higher than the compressive strength of BS-T, and the compressive strength of BS-G also met the needs of bone defect repair. The results of in vitro cell culture tests showed that BS-G had no cytotoxicity. In a Sprague–Dawley rat experimental model, blood tests and key organ sections showed no significant difference between the experimental group and the control group. The prepared BS-G was verified to have good biocompatibility and lays a foundation for the subsequent study of the bone repair effect of radial gradient long bone scaffolds in large animals.

## 1. Introduction

Bones are essential supporting tissues in the human body, playing a pivotal role in the maintenance of normal human activities. Nevertheless, in the United States alone, more than 6.2 million fractures occur each year, with 10% of these cases failing to heal properly [1]. The prevalence of large bone defects caused by fractures, infections, tumors, and systemic diseases has led to an increased demand for bone damage tissue replacement in recent years [2]. This has become a significant and urgent problem in modern clinical medicine, necessitating a multidisciplinary approach to address the major challenges facing the healthcare industry [3]. In clinical practice, surgical treatment (such as bone grafting and bone implants), physical therapy, and drug therapy are commonly used solutions for bone defects [4]. However, these methods face challenges, including the scarcity of bone donors, size mismatch, and immune rejection, which limit their further application [5]. Fortunately, to overcome the above clinical problems, bone repair scaffolds with specific biological properties have been developed based on tissue engineering methods, which can be used to promote the rapid repair of large bone defects.

The emergence of 3D printing technology has enabled the design and manufacture of bone defect repair scaffolds with complex structures and high customization [6]. Combining this technology with tissue engineering, biodegradable 3D porous scaffolds can be prepared, providing new strategies for repairing large bone defects. Although numerous bone tissue engineering scaffolds have been developed, the majority of these prepared scaffolds are relatively simple and regular “well” symmetrical structures. However, it should be noted that natural human bones are a rather uneven tissue from a transverse cross-section. These bones are composed of outer cortical bone and inner cancellous bone [7]. The porosity and pore size of the cortical bone are both small, which increases the rigidity and strength of the bone scaffold and plays a supporting and protective role [8]. The porosity is 30%, the elastic modulus is 4–30 GPa, and the compressive strength is 180–200 MPa. Cancellous bone is a network structure formed by a large number of lamellar trabeculae, with a pore size of 500–1200 microns, a porosity of 55–68%, an elastic modulus of 0.1–2 GPa, and a compressive strength of 0.1–16 MPa [9,10,11]. The porosity and pore size of cancellous bone are larger than those of cortical bone, which allows for the growth of blood vessels and bone marrow, but the compressive properties of cancellous bone are one order of magnitude lower than those of cortical bone [12]. The gradient structure in human natural bones includes the transition from dense cortical tissue to porous trabecular tissue, which is crucial for ensuring the normal function of biological tissues [13].

Bionic functional tissue-like structures have the potential to facilitate the regeneration of bone defects [14]. To meet the varying requirements of bone tissue, the utilization of bionic strategies to design and prepare high-performance bone tissue engineering scaffolds has attracted considerable attention in recent years. A variety of techniques have been employed to fabricate bionic materials, including iterative layering followed by freeze drying [15], layer-by-layer assembly [16], and 3D printing [17]. Functionally graded porous (FGP) structures can be utilized to simulate the structure of natural bone due to their excellent biomimetic morphology and mechanical and biological properties [18]. Among these, functionally graded porous structures are divided into linear and radial types [19,20]. Studies have demonstrated that, in comparison to uniform pore structures, linear gradient and radial gradient functional structures have been shown to enhance mechanical properties [21], cell proliferation [22], and bone formation [23]. This is primarily attributed to the distinct porosities of the different layers of gradient scaffolds. In a previous study, a bidirectional material mixing module based on the structural and component characteristics of natural bone tissue was designed and developed. By varying the content composition of hydroxyapatite, the primary inorganic component of natural bone tissue, in the scaffold [24], a linear graded biomimetic bone scaffold with a gradient ratio of inorganic/organic components was prepared [25]. Its mechanical properties are comparable to those of human bone tissue. In the functionally graded porous structure, the linear gradient structure provides a superior elastic modulus, while the radial gradient structure provides a dense cancellous bone structure with enhanced energy absorption [26]. It is noteworthy that for injuries to large bones such as the tibia, it is even more crucial that the bionic bone scaffold also has radial gradient changes. The creation of a structural gradient in the scaffold pore size can more accurately simulate these regional changes to match the tissue characteristics of cortical bone and cancellous bone [27].

In this study, a radial gradient long bone scaffold with a functionally graded porous structure was manufactured using the dual-phase composite forming process. In comparison with the scaffold produced through conventional techniques, the radial gradient long bone scaffold detailed in this paper exhibits a comparatively dense pore structure on the exterior, accompanied by an elevated concentration of inorganic material hydroxyapatite. This provides the requisite strength and stiffness for bridging bone defects. In the relatively loose interior of the gradient scaffold, organic materials are used to construct a larger pore structure, thereby establishing an optimal osteogenic microenvironment that simulates the structural morphology of cancellous bone in natural bone. In comparison with the traditional scaffold printing process, this study demonstrates the realization of continuous printing of structural gradient and concentration gradient through the precise control of the micropump. The experimental results substantiate the rationale and feasibility of the radial gradient long bone scaffold as a large bone repair material.

## 2. Materials and Methods

### 2.1. Materials

In this study, composite gradient bone scaffolds were fabricated utilizing sodium alginate, gelatin, and nano-hydroxyapatite. Specifically, sodium alginate (SA, 20201110) was sourced from Shanghai Sinopharm Reagent Co., Ltd. (Shanghai, China), while gelatin (Gel, V900863-500G) was obtained from Sigma-Aldrich Trading Co., Ltd. (Shanghai, China). The nano-hydroxyapatite (nHA, 40 nm, needle-like) was procured from China Blue Chemical Company (Dongfang, China). The cross-linking agent employed was anhydrous calcium chloride (CaCl_2_), which was also acquired from the Shanghai Sinopharm Reagent Group. All reagents were utilized in accordance with the manufacturers’ instructions.

### 2.2. Design of Radial Gradient Long Bone Scaffold Model

To fully simulate the structure and function of natural long bones, gradient bone scaffolds must exhibit a gradient change in morphology and composition from the exterior to the interior in the radial direction. In particular, the exterior of the scaffold should exhibit a higher density, while the interior should display a gradual increase in porosity to create a porous structure. Based on the measurement data of human long bones reported previously [28], this study focuses on the study of long bones with a diameter of 30 mm and a height of 3.2 mm as an example to prepare a bone scaffold that matches its size and structure.

**Figure 1:**
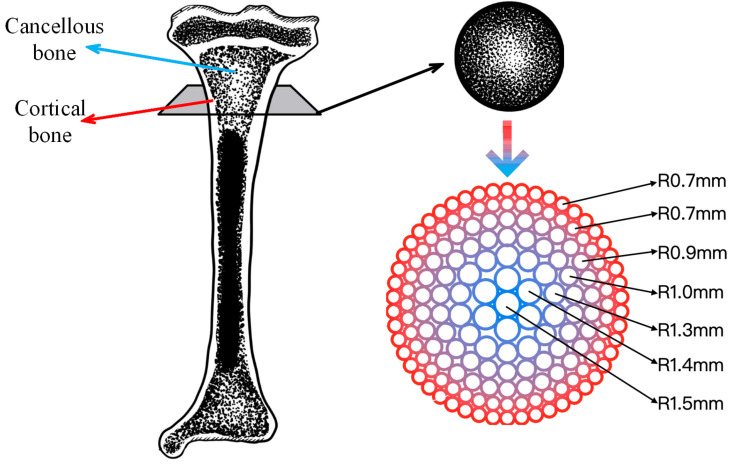
Radial gradient long bone scaffold structure.

To emulate the intricate pore distribution patterns observed within natural long bones, the bone scaffold devised in this study exhibits a gradient in pore structure, whereby the pore size of the scaffold exhibits a continuous change in size. Concurrently, it is essential to maintain an equilibrium between the stiffness of the scaffold and that of the surrounding tissue to prevent the stress shielding effect [29]. The external pore size of the bone scaffold of the long bone studied in this paper is as small as possible to mimic the cortical bone structure, with the pore radius of the outermost circle and the second outer circle being 0.7 mm. The pore radius of the scaffold gradually increases from the external to the internal surface. Additionally, it is important to note that each circle can be completely filled during the scaffold formation process to facilitate continuous printing. Consequently, the distribution of pore radii exhibits a gradual increase from the exterior to the interior, with values of 0.9 mm, 1.0 mm, 1.3 mm, 1.4 mm, and 1.5 mm, respectively, as illustrated in Figure 1.

**Figure 2:**
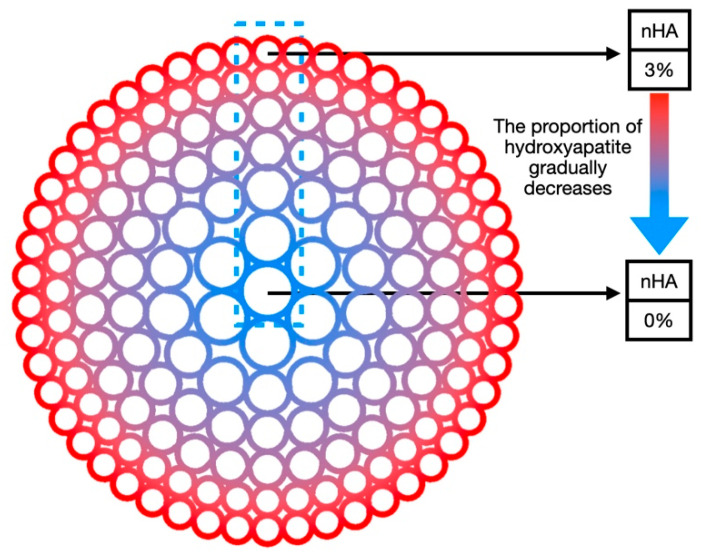
Schematic diagram of radial gradient long bone scaffold composition gradient.

The concentration of the inorganic component nHA in the radial gradient long bone scaffold designed in this study decreases gradually from the outside to the inside along the radial direction. The nHA content decreases from 3% in the outermost part of the scaffold to 0% in the central area (as shown in Figure 2). As the inorganic component decreases, the organic component increases in a manner consistent with the observed trends in natural bone tissue. In this gradient scaffold model, the high concentration of nHA (3%) in the outermost circle provides the scaffold with excellent mechanical properties and plays a supporting role similar to cortical bone. However, the pure hydrogel matrix in the inner central area has low mechanical strength, yet it has better biocompatibility and can provide cells with a suitable proliferation and vascularization microenvironment, thus playing a biological function similar to cancellous bone. The gradient transition zone between the two serves to connect cortical bone and cancellous bone, thereby enhancing the overall performance of the scaffold to a greater degree than would be possible with a single material.

### 2.3. Construction of a Radial Gradient Long Bone Scaffold Forming Platform

**Figure 3:**
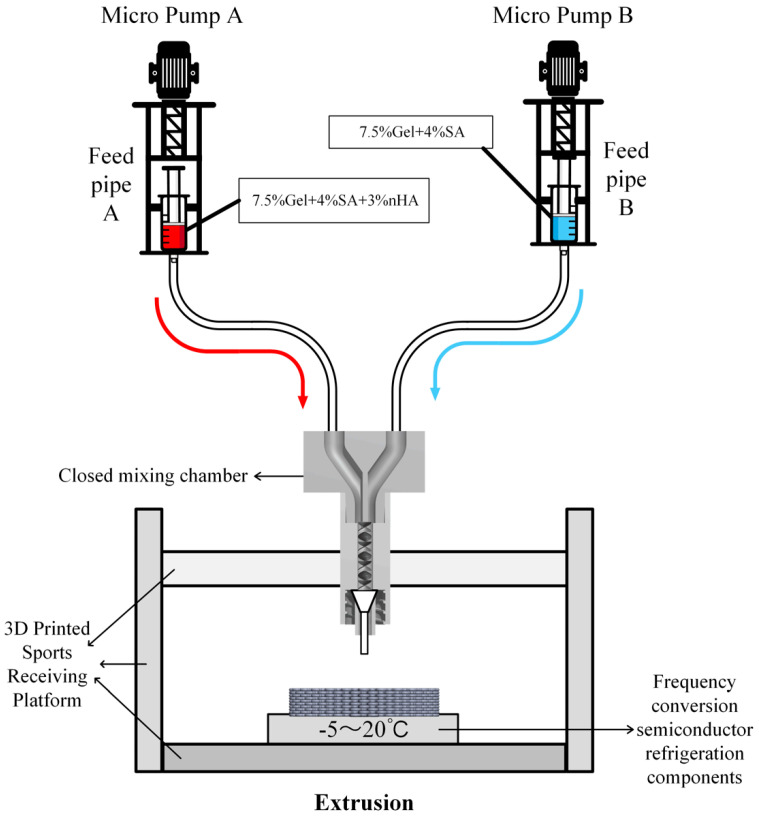
Structure diagram of the test platform for radial gradient long bone scaffold.

This study presents the development of a novel forming equipment platform designed for the fabrication of bionic long bone scaffolds. The objective is to achieve the comprehensive forming process of radial gradient long bone scaffolds, as illustrated in Figure 3.

**Figure 4:**
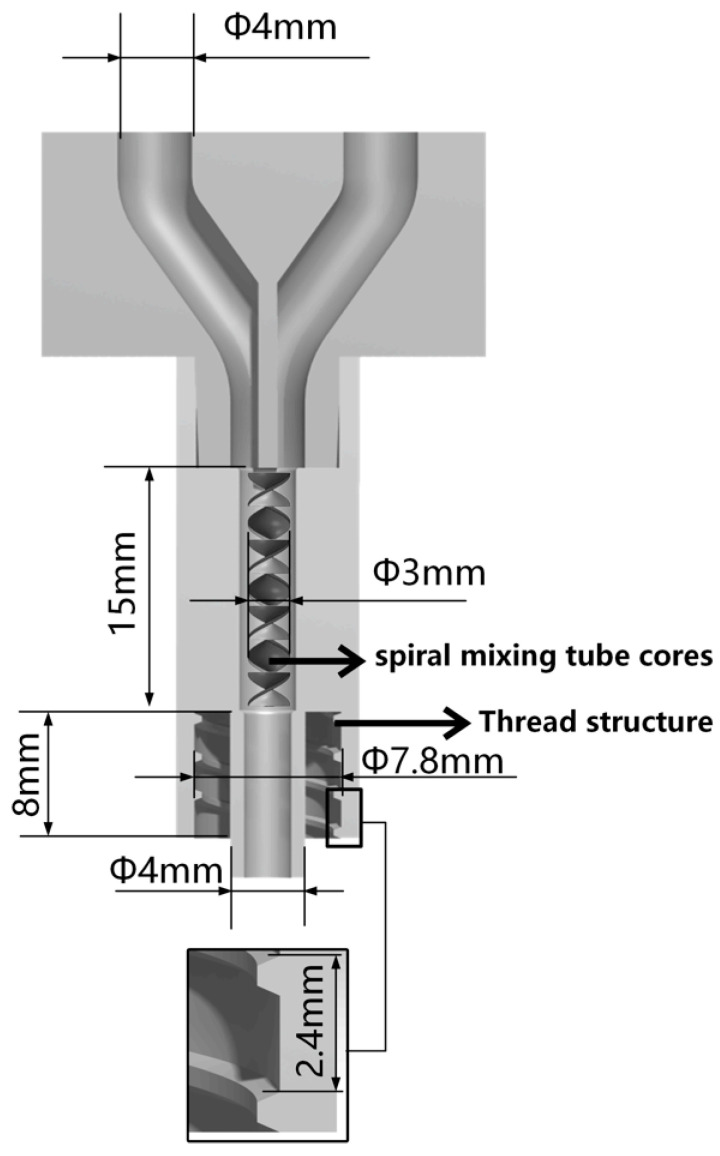
Sectional view of closed mixing chamber.

Micropumps A and B, situated at the left and right extremities of the figure, are operated by the STM32 motherboard and rotate in a forward direction at a predefined velocity, thereby facilitating the delivery of bio-ink printing materials. The micropump employs a lead screw with a diameter and lead of 8 mm and 1 mm, respectively, and is driven by a two-phase stepper motor (model: 42BYGH60). The feed pipe is connected to the closed mixing chamber, which is loaded with the spiral mixer [30], as illustrated in Figure 4. The closed mixing chamber is designed and manufactured with a structure that is independent of any other component. The diameter of the upper feed inlet hole is 4 mm, which is consistent with the size of the feed silicone tube. The interior is composed of a seven-thread structure with a diameter of 3 mm. The uniform mixing of organic and inorganic materials is achieved under the extrusion pressure of the micropump. The extrusion port has an outer diameter of 4 mm and an internal threaded structure, which enables it to be adapted to different types of dispensing extrusion needles to achieve a stable and closed connection. A refrigeration module based on water cooling is installed on the 3D printing motion receiving platform to regulate the temperature of the receiving platform. The temperature sensitivity of gelatin is employed to preclude the collapse of the bracket structure deposited on the platform due to excessive temperature [31], thereby maintaining the integrity and accuracy of the forming process. The refrigeration module primarily comprises essential components, including the refrigeration system, controller, water pipeline, heat exchanger, and water tank. It is operated by a 220 V power supply. The module is programmed to adjust the refrigeration power in real time based on the temperature of the bracket and the number of printed layers. During the refrigeration process, heat is transferred to the water tank via the water pipe, thereby facilitating effective heat exchange and preventing direct impact on the temperature of the bracket receiving platform. This approach ensures that the platform temperature is maintained within an optimal range, which is crucial for ensuring stable forming of the material without expansion due to frost.

### 2.4. Preparation of Radial Gradient Long Bone Scaffold Biomaterial Inks

Biomaterial inks containing varying concentrations of nHA (hereinafter collectively referred to as Gel/SA-X, where X is the nHA content in the biomaterial ink) were prepared for the fabrication of radial gradient long bone scaffolds. Briefly, 0.75 g gel and 0.3 g nHA were dissolved in 10 mL deionized water and stirred on a constant-temperature magnetic stirrer while being heated in a water bath at 50 °C. After 2 h, 0.4 g SA was added to the mixed solution and stirred for 3 h while maintaining the water bath heating temperature at 50 °C. Gel/SA-3 was prepared. Similarly, Gel/SA-0 and Gel/SA-1.5 were prepared in an identical manner to Gel/SA-3, with the exception that the quantities of nHA were 0 g and 0.15 g, respectively. The composition of the biomaterial inks are presented in Table 1.

Calcium chloride (CaCl_2_) was dissolved in deionized water and stirred on a constant-temperature magnetic stirrer until fully dissolved to prepare a 10% (*w*/*v*) CaCl_2_ solution, which was used as a cross-linking agent for biomaterial ink.

### 2.5. Rheological Testing

The rheological properties of Gel/SA-0, Gel/SA-1.5, and Gel/SA-3 were evaluated by using a rotational rheometer (Discovery HR-20, TA Instruments, New Castle, PA, USA). The biomaterial ink was initially positioned in the center of two flat plates with a gap distance of 0.5 mm. The viscosity of the biomaterial inks was tested at constantly increasing shear rates from 1 to 100 s^−1^ at 25 °C. 

### 2.6. Preparation of Radial Gradient Long Bone Scaffolds

BS-0, BS-1.5, BS-3, and BS-G were prepared using a dual-phase composite forming process. BS-0, BS-1.5, and BS-3 were homogeneous bone scaffolds with nHA contents of 0% (*w*/*v*), 1.5% (*w*/*v*), and 3% (*w*/*v*), respectively, while BS-G was a radial gradient long bone scaffold with a gradual change in nHA content along the radial direction. 

**Figure 5:**
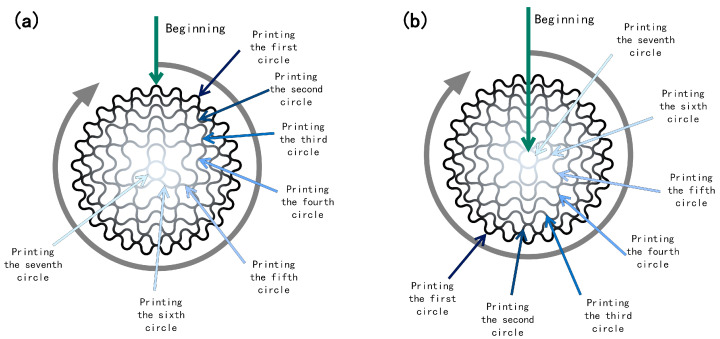
Schematic print paths for odd and even layers: (**a**) odd layer; (**b**) even layer.

The prepared biomaterial ink was loaded into feed pipes A and B, respectively, and then the feed tubes were fixed on the forming platform support frame. At an ambient temperature of 25 °C, the bone scaffolds were prepared in accordance with the key process parameters outlined in Table 2 (the bone scaffold structure is the structure shown in Figure 1). In this study, a 22G needle was utilized, with the Z-axis offset set to 0.27 mm. The dual-phase composite forming process was as follows:

- a. Write G code to control the movement of the 3D-printed receiving platform for each layer of the long bone scaffold, based on the pre-designed radial gradient. Import the printing path G code into the motion controller, and initiate the temperature control module simultaneously, awaiting the receiving platform temperature to decline to the designated value of 8 °C.
- b. Initiate the receiving platform, and, concurrently, micropump A initiates the material in the feed pipe A at a velocity of 0.08 mm/s. The external structure of the radial gradient long bone scaffold is to be formed along the preset path. In order to ensure that the feeding situation matches the movement of the receiving platform, it is necessary to allow for a certain delay in the time for the receiving platform to start moving when printing the first layer.
- c. At the conclusion of the printing of the external structure of the radial gradient long bone scaffold, micropump A ceases to feed, and micropump B commences operation at a speed of 0.08 mm/s. Following the compression of the material within feed tube B into the closed mixing chamber, it is mixed with the remaining material within the closed mixing chamber. Upon completion of the internal structure of the radial gradient long bone scaffold in accordance with the preset path, the gradient ratio of the biomaterial ink composition in the radial direction is achieved, with the innermost circle of the internal structure comprising the material in pure feed pipe B. The nozzle is elevated by 0.27 mm in preparation for the subsequent layer.
- d. To ensure the continuity of the forming path during the forming process, the odd-numbered layer forming path of the radial gradient long bone scaffold commences at the outermost circle of the external structure and terminates at the innermost circle of the internal structure, while the even-numbered layer-forming path is the inverse (Figure 5). Micropump B continues to operate at a speed of 0.08 mm/s to form the internal structure.
- e. Once the internal structure of the radial gradient long bone scaffold has been formed, micropump B is deactivated, and the extrusion process is initiated. The extrusion speed of micropump A is increased to 0.08 mm/s to ensure that when printing the outermost circle of the external structure of the radial gradient long bone scaffold, the material in pure feed pipe A is utilized. Subsequently, the nozzle is elevated by 0.27 mm in preparation for the subsequent layer of printing.
- f. Then, the forming process of the odd-numbered and even-numbered layers of the radial gradient long bone scaffold is repeated in accordance with the aforementioned steps b–e, resulting in a total of eight layers for the radial gradient long bone scaffold.
- g. The radial gradient long bone scaffolds are immersed in a 10% (*w*/*v*) CaCl_2_ solution for 15 min to be cross-linked.

BS-T was the radial gradient long bone scaffold prepared by printing three concentrations of material in separate regions. It was prepared as a control group for comparative mechanical property testing. The aperture structure of BS-T is the same as that of BS-G, both of which have the structure shown in Figure 1. The print path of BS-T is the same as that of BS-G, both of which are shown in Figure 5. However, BS-T achieves the desired gradient distribution of the material through conventional processing techniques. This involves the use of Gel/SA-3 for printing the first and second circles, Gel/SA-1.5 for printing the third and fourth circles, and Gel/SA-0 for printing the fifth, sixth, and seventh circles. 

### 2.7. Morphology Characterization

The morphological characteristics of BS-G were characterized by tungsten filament scanning electron microscopy (SEM, SU1510, Hitachi, Tokyo, Japan). Prior to electron microscopy observation, the dried scaffolds were gold-sprayed using an ion sputtering device to enhance the imaging quality of the electron microscope. Under an accelerating voltage of 15 kV and a high vacuum environment, the surface morphology, porosity changes, and structural integrity of the bone scaffolds were meticulously examined at varying magnifications.

### 2.8. X-ray Powder Diffraction Analysis

Gel, SA, nHA, and BS-G were analyzed by X-ray powder diffraction (XRD, 3KW D/Max-2200, Rigaku, Tokyo, Japan) with the instrument set to monochromatic Cu Kα radiation (k = 1.5405 Å). A speed of 5°/min between 5° and 90° was set to acquire the data.

### 2.9. Mechanical Properties Testing

In order to facilitate a comparative analysis of the mechanical properties of radial gradient long bone scaffolds prepared by the dual-phase composite forming process and that of radial gradient long bone scaffolds prepared by printing three concentrations of material in separate regions, the mechanical properties of BS-1.5, BS-T, and BS-G were evaluated by material testing machine (BIAXIAL-10KN, ZwickRoell, Ulm, Germany). The scaffolds were placed on the material testing machine and subjected to a quasi-static axial compression test at a speed of 2 mm/min. And the resulting data were recorded and analyzed (*n* = 4).

### 2.10. Cell Culture

Human umbilical vein endothelial cells (HUVECs) and C57BL/6 mouse bone marrow mesenchymal stem cells (BMSCs) were obtained from the Cell Bank of the Chinese Academy of Science (Shanghai, China) and Cyagen Biosciences (Guangzhou, China), Inc., respectively. The HUVECs were cultured in high-glucose Dulbecco’s modified Eagle’s medium (DMEM) supplemented with 10% fetal bovine serum (FBS) and 1% penicillin–streptomycin in a humidified atmosphere of 5% CO_2_ at 37 °C, while the BMSCs were incubated in Minimum Essential Medium Eagle-alpha Modification (α-MEM) supplemented with 10% FBS and 1% penicillin–streptomycin. The cells were passaged after trypsinization (0.25% trypsin–EDTA) once they reach 80% confluence.

### 2.11. Cell Proliferation Assay

In order to evaluate the impact of radial gradient long bone scaffolds prepared by dual-phase composite forming process on cell proliferation, the quantitative assays of cell proliferative activity were conducted on BS-0, BS-1.5, BS-3, and BS-G using Cell Counting Kit-8 reagent (CCK-8; Jiangsu KeyGen Bio TECH Co., Ltd., Nanjing, China). The scaffolds were sterilized by immersing in the 75% alcohol in a 24-well plate for 2 h under the UV light, then washed and replaced ethanol using PBS for 3 h. Subsequently, referring to our previous work [4], the scaffolds were immersed in fresh culture medium for 48 h to prepare the scaffold leaching solution. The cell suspension was prepared by the treated culture medium with a density of 1 × 10^5^ cells/mL in accordance with the methodology for preparing cell suspensions during cell passage and added to a 96-well plate with a pipette, 100 μL per well. The cell suspension prepared with pure culture medium served as the positive control group (control). Subsequently, the well plate was placed within a cell culture incubator for the duration of the culture period.

After 1 and 3 days of culture, the 96-well plate was placed on a clean bench. In a light-proof environment, 10 μL of CCK-8 reagent was added to each well, and the plate was then incubated for the specified time (60 min for the HUVECs test and 90 min for the BMSCs test). Subsequently, the well plate was placed in an ELISA reader (infin200Pro, Tecan Group Ltd., Männedorf, Switzerland) to quantify the absorbance of each well at a wavelength of 450 nm. 

### 2.12. Cell Adhesion Assay

The impact of radial gradient long bone scaffolds prepared via dual-phase composite forming process on cell adhesion was evaluated through a comparative analysis of BS-0, BS-1.5, BS-3, and BS-G. The pretreated sterile scaffolds (BS-0, BS-1.5, BS-3, and BS-G) were immersed in fresh culture medium for a period of two hours, after which they were placed in a sterile culture dish. Then, 500 μL of cell suspension with a cell concentration of 1 × 10^6^ cells/mL was evenly distributed on the scaffold. Subsequently, the scaffold inoculated with cells was placed in a constant temperature incubator for a period of four hours. Subsequently, the culture medium was added to the culture dish in which the scaffold was placed, ensuring that the culture medium completely immersed the bone scaffold. Finally, the culture dish was placed in a constant-temperature incubator and the culture medium was replaced every two days.

The mixture was left to stand for 5 min to obtain a stable dye. After one and three days, the cells on the surface of the scaffolds were stained using a live/dead cell staining kit (MX3012-500T, Maokangbio, Shanghai, China) in a dark environment. This was then placed in a constant-temperature incubator for a period of 15 min. Subsequently, the bone scaffold was placed on a glass slide. To observe the cells on the surface of the scaffolds, an inverted fluorescence microscope (LHM100CB-1, Nikon, Tokyo, Japan) was used to observe the live and dead cells.

### 2.13. Subcutaneous Embedding Test

In the evaluation of in vivo biocompatibility of radial gradient long bone scaffolds prepared by dual-phase composite forming process using BS-G, male Sprague–Dawley (SD) rats, aged 6 weeks, were used in the experiment, and all animal experiments received approval from the Ethics Committee of Shanghai University (Approval No. ECSHU 2024–022). The SD rats were anaesthetized with 2% (*w*/*v*) sodium pentobarbital (40 mg/kg). The dorsal surface was shaved and disinfected with 75% ethanol, and a full-thickness wound (30 × 30 mm) was incised. The disinfected BS-G was then attached to the wound surface, and the epidermis was sutured. The control group was served as the one in which the epidermis was sutured without inserting any scaffolds. Two weeks following scaffold implantation, blood samples were collected from the rats for hematological and biochemical analysis. This was carried out to study the potential in vivo toxicity of radial gradient long bone scaffolds. Subsequently, three rats from each group were euthanized for analysis. The main organs (heart, liver, spleen, lung, and kidney) of the rats were excised and preserved, and hematoxylin and eosin (H&E) staining were employed for histological examination.

### 2.14. Statistical Analysis

All data are expressed as mean ± standard deviation, and one-way analysis of variance was used to determine which populations had significant differences. *p* < 0.05 was considered as significant difference. Statistical analysis was performed using Origin 2022 software.

## 3. Results

### 3.1. Printing Performance Verification of Radial Gradient Long Bone Scaffolds

**Figure 6:**
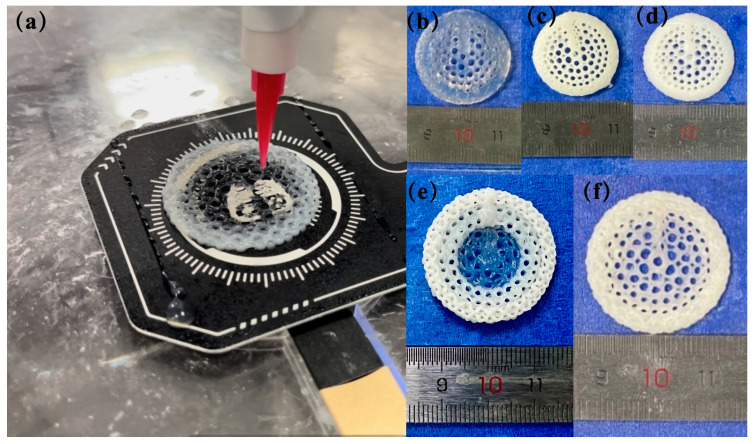
Radial gradient long bone scaffold formation: (**a**) preparation process of radial gradient long bone scaffold BS-G; (**b**–**f**) physical images of physical images of the scaffold.

During the preparation process, the temperature of the 3D printing motion receiving platform was carefully controlled to form a radial gradient long bone scaffold. The scaffold material did not collapse, and the structure remained intact and stable. The fiber filaments exhibited uniformity and continuity (Figure 6a). The formation effects of each group of scaffolds are illustrated in the accompanying figure (Figure 6b–f). The five scaffolds were of a similar structure, exhibiting a gradient distribution of pore sizes. The three groups of scaffolds, BS-0, BS-1.5, and BS-3, demonstrated homogeneous characteristics. The nHA content within these scaffolds was 0% (*w*/*v*), 1.5% (*w*/*v*), and 3% (*w*/*v*), respectively. As the concentration of nHA in the homogeneous scaffolds of BS-0, BS-1.5, and BS-3 increased, the transparency of the scaffold color decreased. From the outermost circle to the innermost circle of BS-G, the transparency of the fiber color exhibited a gradual increase, indicating that the concentration of nHA in BS-G decreased radially from the outermost circle to the innermost circle, exhibiting a gradient distribution. In contrast to the BS-G preparation method, the BS-T approach entailed the utilization of three distinct biomaterial inks, each employed for a specific region of the printed scaffolds. As illustrated in the Figure 6e, BS-T contained 3% (*w*/*v*) nHA in the first and second circle, which exhibited the lowest transparency. In contrast, the third and fourth circle contained 1.5% (*w*/*v*) nHA and displayed increased transparency. The fifth, sixth, and seventh circle were devoid of nHA and exhibited the highest transparency. A distinct chromatic differentiation was evident between the various components. The boundaries between the constituent parts of the bracket were clearly discernible. 

### 3.2. Analysis of Rheological Properties

**Figure 7:**
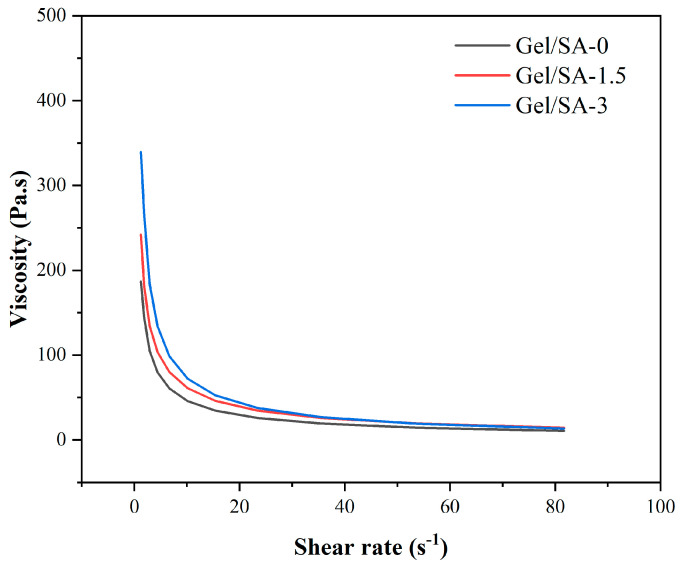
Viscosity as a function of shear rate at 25 °C.

Figure 7 illustrates the viscosity of Gel/SA-0, Gel/SA-1.5, and Gel/SA-3. These data suggest that that an increase in nHA content resulted in a corresponding rise in the viscosity of the biomaterial inks, a finding that was corroborated by the extant literature. The viscosity of all three biomaterial inks diminished with the elevation in shear rate, exhibiting shear-thinning characteristics. Therefore, the three biomaterial inks, Gel/SA-0, Gel/SA-1.5, and Gel/SA-3, were suitable for the forming process [32].

### 3.3. XRD Analysis

**Figure 8:**
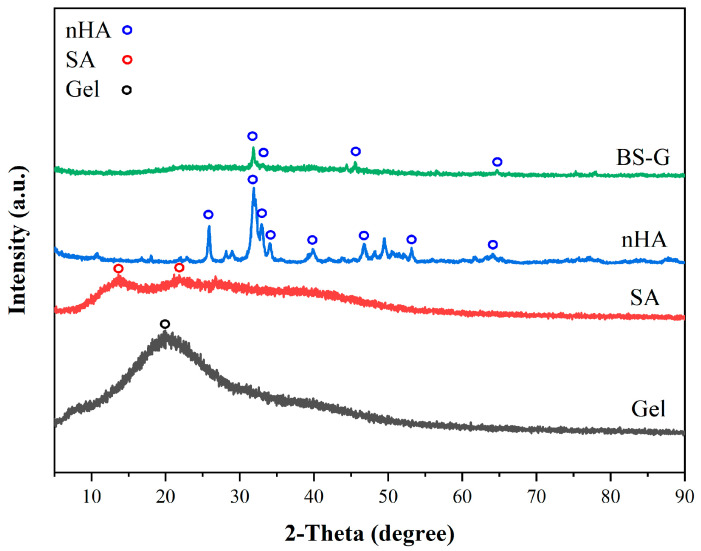
The XRD patterns of Gel, SA, nHA, and BS-G.

Figure 8 illustrates the XRD patterns of Gel, SA, nHA, and BS-G. The gel was known in the non-crystalline state, with broad diffraction peaks at 2θ = 20° [33]. SA displayed two broad diffraction peaks at 2θ = 13.6° and 2θ = 21.8°, which related to the reflection of the plane from the polyguluronate unit and the plane from the polymannuronate unit in the SA [34]. nHA displayed some characteristic diffraction peaks at 2θ = 25.9°, 31.8°, 33°, 34°, 39.8°, 46.8°, 53.1°, and 64.1°, which was in agreement with the standard card for nHA (JCPDS-09–0,432). The presence of several diffraction peaks of nHA in BS-G is likely attributable to the encapsulation of hydroxyapatite within a gelatin and sodium alginate matrix.

### 3.4. Morphology Characterization 

**Figure 9:**
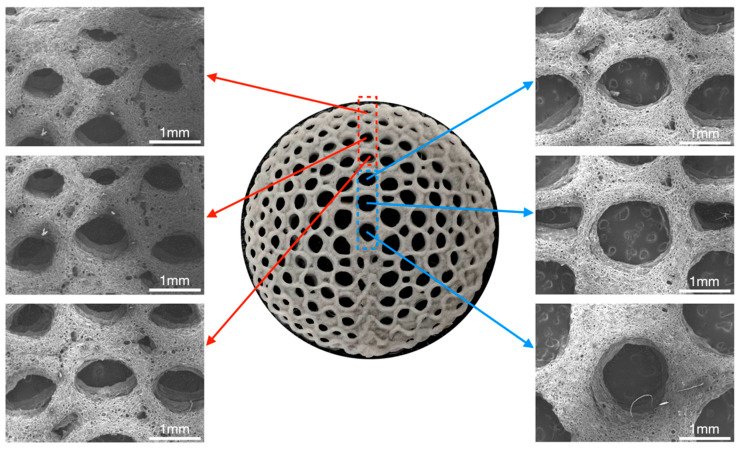
Macroscopic and microscopic morphology of radial gradient long bone scaffold.

The structures of the radial gradient long bone scaffold, designated BS-G, are depicted in Figure 9. This figure presents structural images of the radial gradient long bone scaffold, captured from the exterior to the interior, at a magnification of 32 times under a scanning electron microscope. The pore size of the macroscopic pores of the BS-G scaffold gradually increases from the outermost circle to the innermost circle, exhibiting a clear structural gradient. This differs from the pore size of the radial gradient long bone scaffold originally designed. The primary reason for this phenomenon is that the hydrogel undergoes slight deformation following complete drying, which in turn affects the shape of the macroscopic pores. 

### 3.5. Mechanical Properties Test Analysis

**Figure 10:**
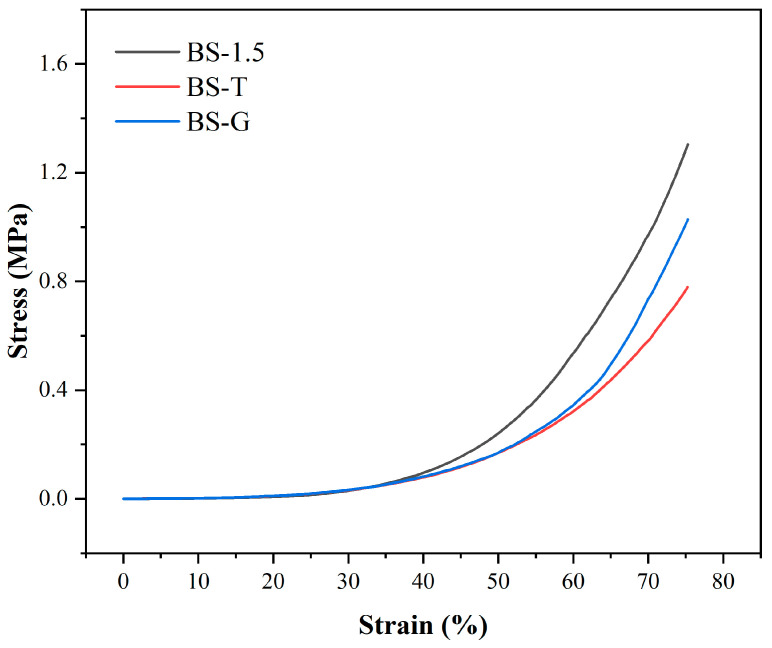
Compressive stress–strain curve.

Figure 10 illustrates the compressive stress–strain curve of the radial gradient long bone scaffold. Upon the radial gradient scaffold to a strain of 75%, the strain of BS-G exhibited a slight decline in comparison to that of the BS-1.5. As illustrated in Table 3, when the BS-G scaffold was subjected to 75% strain, it exhibited a compressive stress of 1.00 ± 0.19 MPa. Nevertheless, BS-G with continuous gradient tapering exhibits superior mechanical properties in comparison to BS-T with gradient scaffolds prepared through the conventional process. According to reports, the reference range of compressive stress for natural human bone to be repaired is 1–10 MPa [35]. Consequently, the mechanical properties of the radial gradient bone scaffold prepared in this study are sufficient for the repair of bone defects.

### 3.6. Analysis of Cell Proliferation Assay

**Figure 11:**
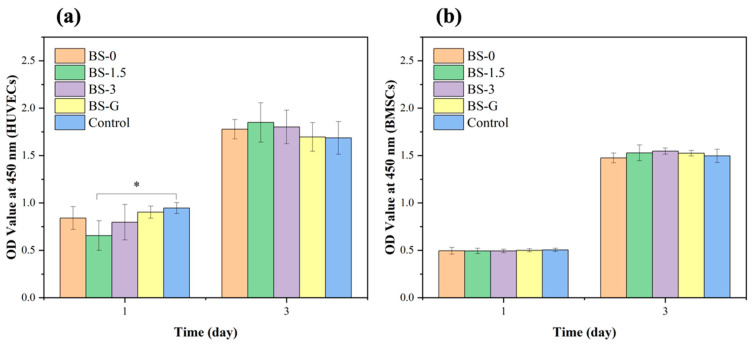
Proliferation test results of scaffolds in each group: (**a**) HUVEC proliferation test results; (**b**) BMSC proliferation test results.

The CCK-8 test results of HUVECs and BMSCs cultured in the scaffold leaching solution are presented in Figure 11. During the three-day culture period, the OD values at 450 nm of the BS-0, BS-1.5, BS-3, and BS-G groups were comparable to those of the control group in terms of the growth trends of HUVECs and BMSCs. Furthermore, there was no significant difference between the OD value of the BS-G group and the control groups. The cells cultured in the radial gradient scaffold leaching solution exhibited normal proliferation, which preliminarily indicated that the radial gradient scaffold had no adverse effect on the proliferation ability of HUVECs and BMSCs. This indirectly proved that the scaffold had good biocompatibility.

### 3.7. Analysis of Cell Adhesion Assay

**Figure 12:**
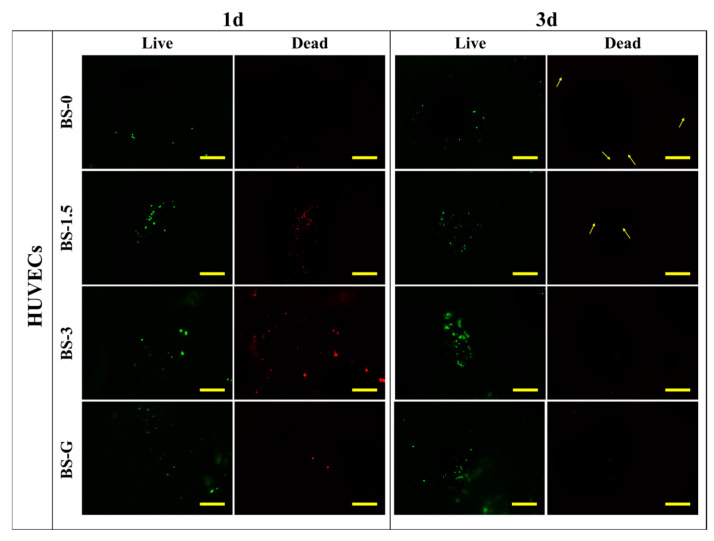
HUVEC adhesion test results (scale bar is 500 μm).

**Figure 13:**
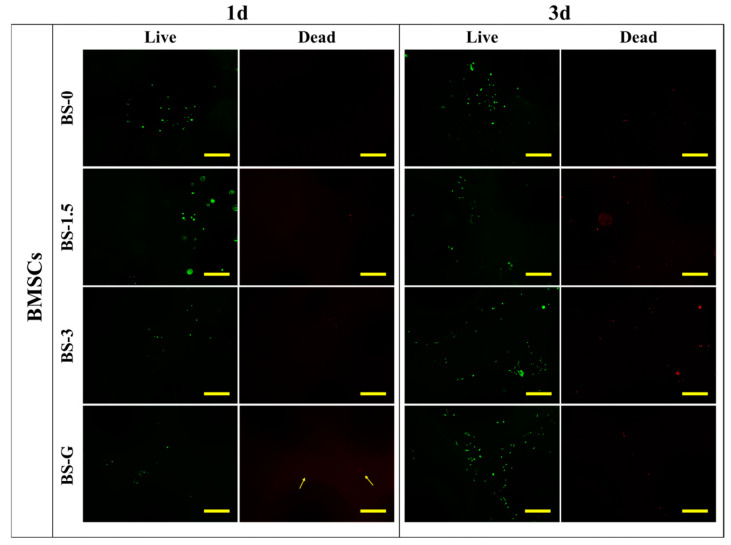
BMSC adhesion test results (scale bar is 500 μm).

Figure 12 and Figure 13 illustrate the staining images of live and dead cells on the surface of radial gradient bone scaffolds. On the first day, a small number of HUVECs and BMSCs adhered to the surface of the scaffold. After the third day, the HUVECs and BMSCs began to proliferate on the surface of the scaffold. These findings are consistent with the results of cell proliferation detection. On the first and third days, there were dead cells of HUVECs and BMSCs on the surface of the scaffold. However, as the cells on the surface of the scaffold began to proliferate on the third day, although the number of dead HUVECs also increased, the number of dead cells increased far less than that of live cells. The test results demonstrate that the radial gradient long bone scaffold has no obvious cytotoxicity, thereby corroborating its biocompatibility.

### 3.8. In Vivo Bioassay Analysis

**Figure 14:**
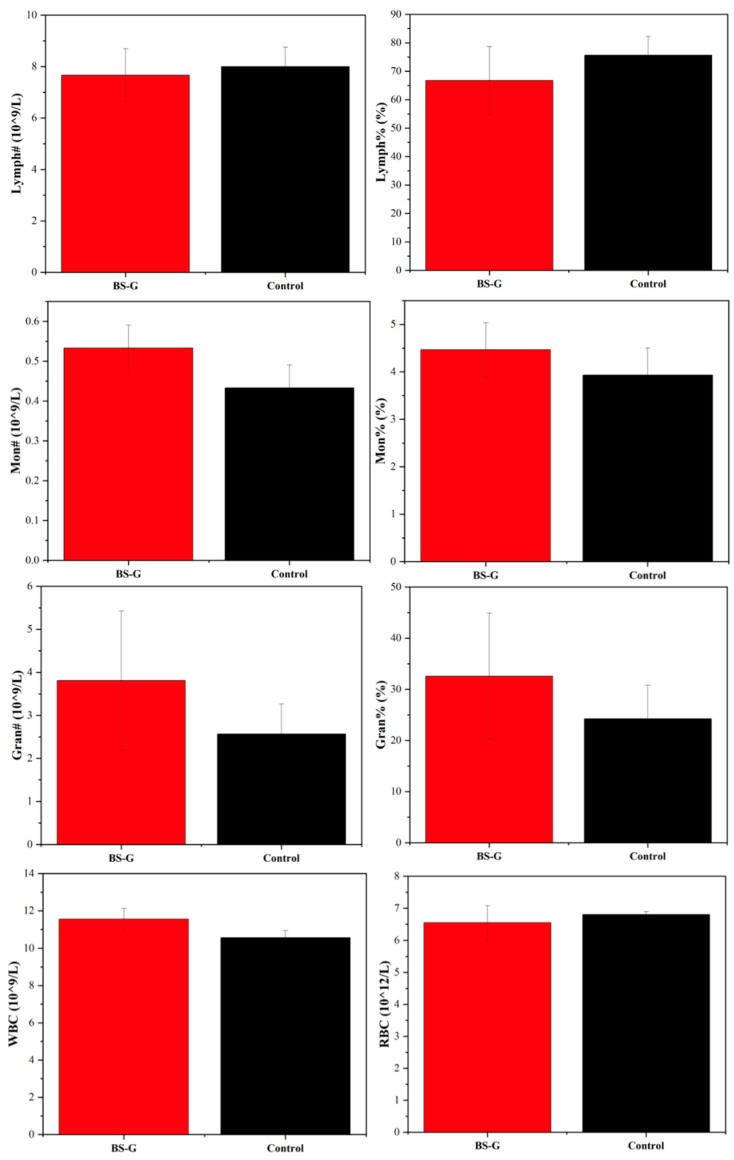
Blood routine test results.

**Figure 15:**
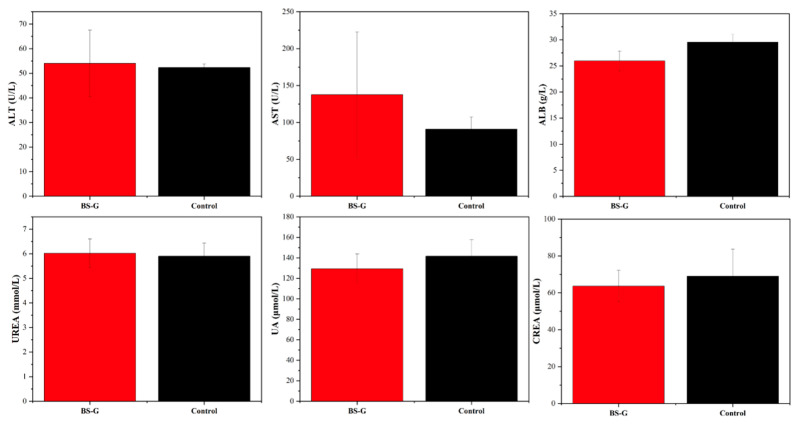
Results of liver and kidney function biochemical tests.

Although in vitro studies have preliminarily validated the biological properties of scaffolds, it is particularly important to study the biocompatibility of scaffolds in real biological environments through in vivo experiments. The results of the rat blood routine tests are presented in Figure 14. The results indicate that the biomaterial ink does not induce inflammation, as evidenced by the normal counts and percentages of white blood cells (WBC), lymphocytes (LYM), monocytes (MON), and neutrophils (N). Furthermore, there were no discernible alterations in the rats’ biochemical test results in Figure 15, which pertained to markers associated with hematopoiesis (red blood cell: RBC), liver function (alanine aminotransferase: ALT; aspartate aminotransferase: AST; albumin: ALB), and kidney function (UREA; urine acid: UA; creatinine: CREA). These findings suggest that the radial gradient long bone scaffold does not elicit an immune response.

**Figure 16:**
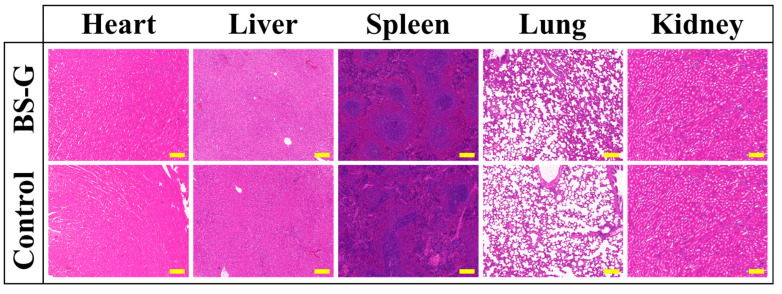
H&E staining results (scale bar is 200 μm).

Following the two-week period during which the radial gradient long bone scaffold was embedded in the rat’s skin, a histological examination of the main organs was conducted using H&E staining. As illustrated in Figure 16, the histological examination of the key organ tissue sections from the experimental group of rats revealed no significant differences in staining results when compared to those of the control group. The histological examination of the tissue sections yielded results that were consistent with those of the blood tests, thereby providing further evidence that the radial gradient long bone scaffold has excellent biocompatibility.

## 4. Discussion

The current research on bone scaffold structure primarily concerns itself with the effects of porosity, pore size, and structural shape on the mechanical properties and bioactivity of the scaffold. Bone scaffolds with a single structure and composition are challenging to create in a way that meets the diverse mechanical properties and microenvironment requirements of different parts of the bone scaffold during bone defect repair. Currently, gradient bone scaffolds that emulate the structural characteristics of natural bone are predominantly manufactured through processes such as extrusion molding [36] and selective laser melting [37]. Among these, extrusion bio-3D printing based on biomaterials offers a wide range of molding material selection [38], the capacity to carry growth factors and cells, and the ability to change the material concentration ratio, growth factors, and cells in a gradient manner as needed, thus meeting the diverse requirements of different materials for different parts of the bone scaffold during bone defect repair [39,40]. The aforementioned characteristics have attracted the attention of researchers, yet the existing process still presents several challenges. These include a discontinuous printing process, uneven material mixing, difficulty in achieving structural gradient changes, and a complex preparation process, which collectively make it difficult to achieve the integrated preparation of radial bionic gradient bone scaffolds.

This study presents a process for the integrated formation of a structural–material gradient. The process involves combining the principal components of natural bone tissue with the performance standards of the requisite bone scaffold. As a natural polymer with analogous biological characteristics to collagen, gelatin not only exhibits superior forming and processing properties, but also displays enhanced mechanical attributes. However, GEL exhibits notable temperature-dependent characteristics, displaying a substantial change in viscosity across different temperature ranges [41]. This property can influence the stability of the printed scaffold structure. To address this limitation, this article introduces SA as a thickener and utilizes its exceptional adhesion properties [42], thermal stability, and gelation ability to develop a SA/GEL composite system. This approach enables the creation of a biological scaffold with favorable biological properties and compatibility, while maintaining high processing stability and mechanical properties. Consequently, the organic phase material of the biological scaffold is an SA and GEL composite hydrogel. nHA is a bioceramic material that has been extensively utilized, particularly in the domain of bone tissue engineering. nHA exhibits robust biological activity and osteogenic guidance, facilitating the growth of bone tissue. Due to its nanoscale particle size, it has a higher specific surface area than ordinary hydroxyapatite, which allows for greater adsorption of cells and proteins on its surface [43]. By compounding with polymers or other bioceramic materials, its mechanical properties and biological activity can be enhanced. Consequently, nHA is incorporated into it as the inorganic phase. CaCl_2_ solution is selected as the cross-linker for the final stable formation of the scaffold. A theoretical model of a radial gradient long bone scaffold was constructed, and a forming technology scheme integrating structural and material gradients was proposed based on it. The technical solution is proposed for the continuous extrusion of materials with varying concentration gradients from a single nozzle. This is achieved by controlling the extrusion rate of two barrels into a closed mixing chamber. In contrast to the conventional cross-grid structure, the radial gradient long bone scaffold is formed by the interlocking of curves. Consequently, during the printing of radial gradient long bone scaffolds, the fibers are situated in a continuous trajectory, traversing from the outer ring to the center and subsequently from the center to the outer ring (Figure 5). Consequently, in conjunction with the process proposed in this work, a continuous distribution of pore structure gradients and material concentration gradients in the radial direction can be achieved. As illustrated in Figure 6f, the BS-G exhibits excellent overall forming quality, and the seamless continuous printing of the dual gradient of structure and composition along the radial direction of the scaffold is achieved, thereby demonstrating the feasibility of the constructed forming platform and printing process route, as well as the rationality and practicality of the optimized parameter combination. 

To comprehensively evaluate the performance of the prepared radial gradient long bone scaffold, this study employed a range of characterization methods to systematically analyze and test the morphological characteristics and physical and mechanical properties of the scaffold. From the outermost to the innermost circle of BS-G, the transparency of the fiber color exhibited a gradual increase, indicating that the concentration of nHA in BS-G decreased radially from the outermost circle to the innermost circle, exhibiting a gradient distribution. In contrast to the BS-G preparation method, the BS-T approach employs the use of three distinct biomaterial inks, with each ink being utilized for a specific region of the printed scaffolds. As illustrated in Figure 6e, the BS-T preparation contains 3% nHA in the first and second circles, which exhibit the lowest transparency. In contrast, the third and fourth circles contain 1.5% nHA and display increased transparency. The fifth, sixth, and seventh circles are devoid of nHA and exhibit the highest transparency. A notable chromatic differentiation is evident between the various components. The boundaries between the constituent parts of the bracket are clearly discernible. During the processing of BS-T, it has to change the nozzle in order to complete the change in material concentration. This results in the fiber filaments becoming discontinuous at the point where the nozzle changes. Concurrently, there is a precipitous alteration in the material concentration between the constituent parts of the BS-T, which facilitates their separation. The mechanical properties of BS-T are adversely affected. This should elucidate the reasons behind the inferior mechanical properties of BS-T in comparison to those of BS-G.

Furthermore, to verify the biocompatibility of the scaffold, this study also conducted in vitro cell tests and in vivo implantation tests in rat models. In cellular assays, we demonstrated that BS-G is not cytotoxic for BMSCs. Furthermore, the impact of BS-G on HUVECs was examined, as the initial vascularization phase represents a pivotal stage in the osteogenic repair of bone defects [44,45]. This process is contingent upon the involvement of HUVECs, which are capable of secreting bone morphogenetic proteins and facilitating the osteogenic differentiation of BMSCs [45]. Additionally, the experimental results demonstrate that BS-G is non-cytotoxic to HUVECs.

Two weeks following the implantation of BS-G into the rats, blood tests were conducted, and sections of the heart, liver, spleen, lungs, and kidneys were obtained. The results of the tests demonstrated that there was no statistically significant difference between the results of the experimental group and the control group. The preliminary verification from the tests indicates that BS-G exhibits good biocompatibility. Furthermore, Gel, SA, and nHA have been extensively demonstrated to possess favorable biocompatibility [46,47,48,49,50]. This also provides indirect evidence that BS-G has good biocompatibility, which is consistent with the results of in vitro cell culture and in vivo rat tests.

This study verified the rationality of the radial gradient long bone scaffold model and the feasibility of the preparation process. Additionally, the biocompatibility of the scaffold was initially investigated. However, further study is required to fully elucidate the in vivo bone defect repair effect of the radial gradient long bone scaffold. Subsequently, in order to more comprehensively evaluate the osteogenic properties and bone defect repair ability of the prepared radial gradient long bone scaffold, we intend to conduct in vivo experiments in large animal models (such as pigs and cattle). The implantation of the radial gradient long bone scaffold into the bone defect site of large animals would allow for the observation and evaluation of the bone tissue healing process over an extended period of time. In addition, the size and gradient variations of the holes in the bone scaffold must be tailored to the individual bone defect in specific applications. It is therefore necessary to investigate the appropriate pore gradient distribution and material concentration gradient changes for different long bone defect situations in order to obtain improved mechanical and biological properties. Subsequently, pertinent mathematical models will be formulated with the objective of establishing reference points for the design of bone scaffolds for the repair of long bone defects in future clinical applications.

## 5. Conclusions

The objective of this study was to propose a radial gradient bone scaffold preparation process with bionic natural bone properties for the treatment of long bone defects. This process combines multi-material composite and 3D printing technology, enabling the continuous gradient change in structure and composition by controlling the printing path and material extrusion speed. We also developed a corresponding forming platform. The principal advantage of this process is that the structure and material composition of the bone scaffold can be controlled to achieve a closer approximation to the characteristics of natural bone tissue. To evaluate the feasibility and advantages of the radial gradient long bone scaffold preparation process, a series of performance tests were conducted on the prepared radial gradient long bone scaffold, including morphology characterization, compressive strength, and cell proliferation. Finally, in vivo animal tests were conducted based on rat models. The test results demonstrated that BS-G exhibited the anticipated gradient effect, exhibiting morphological characteristics and composition comparable to those of natural bone tissue, whereas BS-T displayed a discontinuous gradient with distinct boundaries between the constituent parts. Moreover, the compressive stress of BS-G is 1.00 ± 0.19 MPa, which is superior to that of BS-T, and its mechanical properties meet the needs of bone defect repair. The results of the in vitro cell culture tests demonstrated that BS-G exhibited no evidence of cytotoxicity. In the SD rat experimental model, no significant differences were observed between the experimental and control groups with regard to blood tests and key organ sections. The prepared BS-G scaffold was found to have good biocompatibility, thus providing a foundation for the subsequent study of the bone repair effect of radial gradient long bone scaffolds in large animals.

## Figures and Tables

**Table 1:** Content of each component of Gel/SA-X biomaterial ink.

Biomaterial Inks	Gel (*w*/*v*)	SA (*w*/*v*)	nHA (*w*/*v*)	Deionized Water (mL)
Gel/SA-0	7.5%	4%	0%	10
Gel/SA-1.5	7.5%	4%	1.5%	10
Gel/SA-3	7.5%	4%	3%	10

**Table 2:** Key process parameters for the preparation of bone scaffold.

Bone Scaffold	Biomaterial Ink (Feed Pipe A, Feed Pipe B)	Material Temperature (°C)	Receiving Platform Temperature (°C)	Feeding Speed (mm/s)	Printing Speed (mm/min)
BS-0	Gel/SA-0 Gel/SA-0	25	8	0.08	400
BS-1.5	Gel/SA-1.5 Gel/SA-1.5	25	8	0.08	400
BS-3	Gel/SA-3 Gel/SA-3	25	8	0.08	400
BS-G	Gel/SA-3 Gel/SA-0	25	8	0.08	400

**Table 3:** Stress of each group of scaffolds at 75% strain.

Scaffold	Stress (MPa)
BS-1.5	1.38 ± 0.49
BS-T	0.96 ± 0.38
BS-G	1.00 ± 0.19

## Data Availability

The data that support the findings of this study are available on request from the corresponding author. The data are not publicly available due to privacy.
